# Qualitative and quantitative assessment of accelerated liver diffusion-weighted imaging using deep-learning reconstruction in oncologic patients

**DOI:** 10.1186/s12880-025-02030-3

**Published:** 2025-11-26

**Authors:** Mihaela Rata, Francesca Castagnoli, Joshua Shur, Emily Evans, Georgina Hopkinson, Thomas Benkert, Elisabeth Weiland, Dow-Mu Koh, Jessica M. Winfield

**Affiliations:** 1 Department of Radiology, MRI Unit, The Royal Marsden NHS Foundation Trust, London, UK; 2 Division of Radiotherapy and Imaging, The Institute of Cancer Research, London, UK; 3 Research and Clinical Translation, Magnetic Resonance, Siemens Healthineers AG, Erlangen, Germany; 4 Royal Marsden NHS Foundation Trust & Institute of Cancer Research, Downs Road, Sutton, London, SM2 5PT, UK

**Keywords:** Liver, Metastasis, Diffusion magnetic resonance imaging, Deep learning reconstruction

## Abstract

**Background:**

Deep-learning (DL) reconstructions could improve image quality and reduce acquisition time in diffusion-weighted imaging (DWI). This study assessed, qualitatively and quantitatively, DL-DWI in liver metastasis of colorectal cancer patients.

**Methods:**

This prospective study enrolled 50 participants from June to November 2022. Phantom and participant data were acquired on a 1.5T MR scanner using a free-breathing DL-DWI research application sequence. Three DWIs were compared: a moderately-accelerated DL-DWI (DL-1), a corresponding standard reconstruction (Standard-1) and a highly-accelerated DL-DWI (DL-2). Image quality (four features on b750 images and one feature on ADC map) was assessed by two radiologists. Region of interest (ROI) based ADC measurements were performed at three locations: liver, spleen, liver metastasis. Across the three series, median scores and ADC values were assessed using a Friedman non-parametric test and post-hoc analysis (pairwise Wilcoxon tests with Bonferroni correction). A p-value < 0.05 was considered statistically significant.

**Results:**

Fifty participants with metastatic colorectal cancer (mean age 62 years, range 36–88 years, 26 males) were evaluated. ROIs were delineated in liver (*N* = 50), spleen (*N* = 48), and liver metastasis (*N* = 11). Qualitatively, across both readers, DL-1 method received the highest scores for 5/8 features on the b750 images; all methods scored similarly on ADC maps for both readers. Quantitatively, ADCs were significantly different between DL-1 and Standard-1 series across all three organs, with DL-1-based ADC always higher (*p* < 0.01). This ADC increase was small: 8.9% (liver), 3.4% (spleen), 4.5% (liver metastasis).

**Conclusions:**

This study suggests that a DL-based reconstruction is a promising technique to enable acceleration of liver DWI considering both qualitative and quantitative results.

**Trial registration:**

NCT05118555 (Evaluation of New Magnetic Resonance Techniques); study date of registration (first submitted: 2021-10-18).

**Supplementary Information:**

The online version contains supplementary material available at 10.1186/s12880-025-02030-3.

## Background

Diffusion-weighted imaging (DWI) is a key component of liver MRI, being used for the detection and characterisation of focal liver lesions [1] and assessment of treatment response [2]. Its associated apparent diffusion coefficient (ADC) map can help differentiate malignant lesions (demonstrating lower ADC values) from normal hepatic tissue or benign lesions [3] and the monitoring of treatment effects (which show increasing ADC values).

However, conventional free-breathing liver DWI has relatively long acquisition times and low signal-to-noise ratio (SNR) due to its relatively long echo times (TE). To date, a common DWI acquisition based on a free-breathing method with multiple signal averages per b-value requires typically ~ 5 min to perform [4]. In addition, respiratory and cardiac motion can also degrade image quality, especially over the left hepatic lobe. Imaging optimisation suggests using the shortest possible TE, as well as parallel imaging techniques to improve SNR [5].

Recent developments in deep-learning (DL) reconstruction methods for DWI might allow reduction in acquisition times whilst maintaining good image quality. Accelerated DWI with DL reconstruction has been assessed lately in several organs such as brain [6], breast [7], prostate [8] or liver [9–13]. In the liver, only three recent studies [10, 11, 13] reported observations on ADC measurements in a patient cohort. A retrospective study from Bae et al. [10] compared respiratory-triggered DWI with DL reconstruction (and higher acceleration factor and lower averages) versus a separate acquisition of respiratory-triggered DWI with standard reconstruction. In a prospective study, Chen et al. [11] reported a direct comparison of respiratory-triggered DWI with and without DL reconstruction. Kim et al. [13] evaluated DL reconstruction in the context of motion compensation strategies by comparing free-breathing DL-DWI versus respiratory-triggered DL-DWI, and respiratory-triggered standard DWI.

Our prospective study assessed, both qualitatively and quantitatively, accelerated DWI with DL reconstruction in the liver by comparing three free-breathing DWI series: a moderately-accelerated DWI with DL reconstruction (DL-1), a corresponding standard reconstruction of the same data (Standard-1) and a highly-accelerated DWI with DL reconstruction (DL-2).

## Methods

This study includes data from patients and a test object.

### Study population

#### Participants

**Fig. 1:**
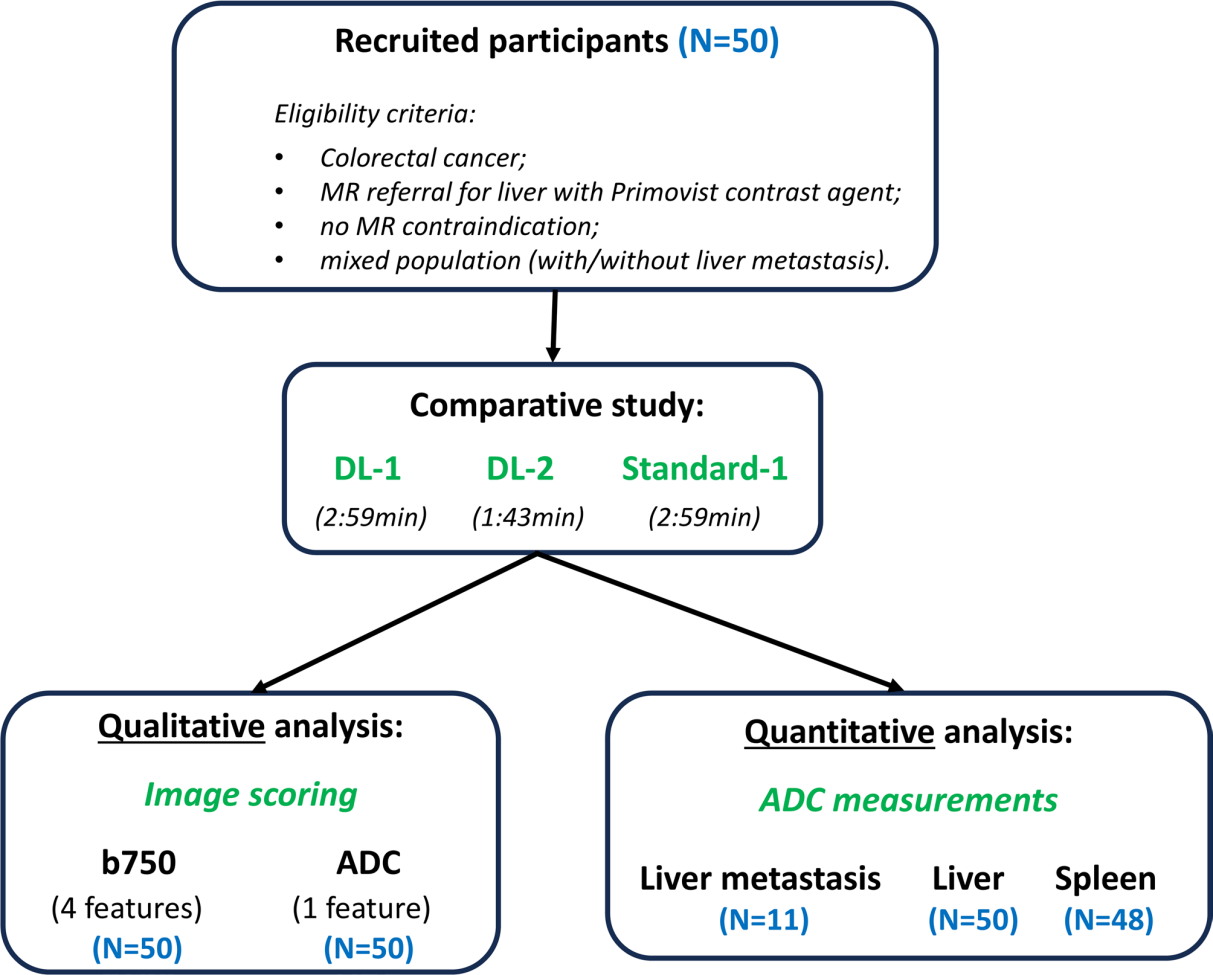
Flow chart of the participants cohort and their subcohort split for further analyses. DL-1 = moderately-accelerated DWI using a deep-learning reconstruction algorithm; DL-2 = highly-accelerated DWI using a deep-learning reconstruction algorithm; Standard-1 = moderately-accelerated DWI using a standard reconstruction algorithm. ADC = apparent diffusion coefficient

From June to November 2022, 50 consecutive participants (mean age 62 years, range 36–88 years, 26 males) were enrolled into a prospective study approved by a national research ethics committee (Table 1; Fig. 1). The study was conducted in accordance with the Declaration of Helsinki. Verbal informed consent was obtained from each participant for the addition of two research sequences (total 5 min) at the end of their clinical examination. The study included participants with colorectal cancer that were referred (for disease staging or follow-up) for clinical liver MR examinations with administration of a hepatobiliary-specific contrast agent (Primovist/Eovist). The cohort included participants at any stage of treatment. Although power calculation found that 16 participants would allow the detection of an effect size of 0.8 in image quality score with 90% power and two-sided alpha = 0.1, we recruited 50-participants to obtain a greater range of examples.

**Table 1:** Demographics of the participants to this study

Cohort	Participants with colorectal cancer
Size	50
Gender	26 males, 24 females
Age (mean ± standard deviation)	62 ± 14 years

#### Test object

**Fig. 2:**
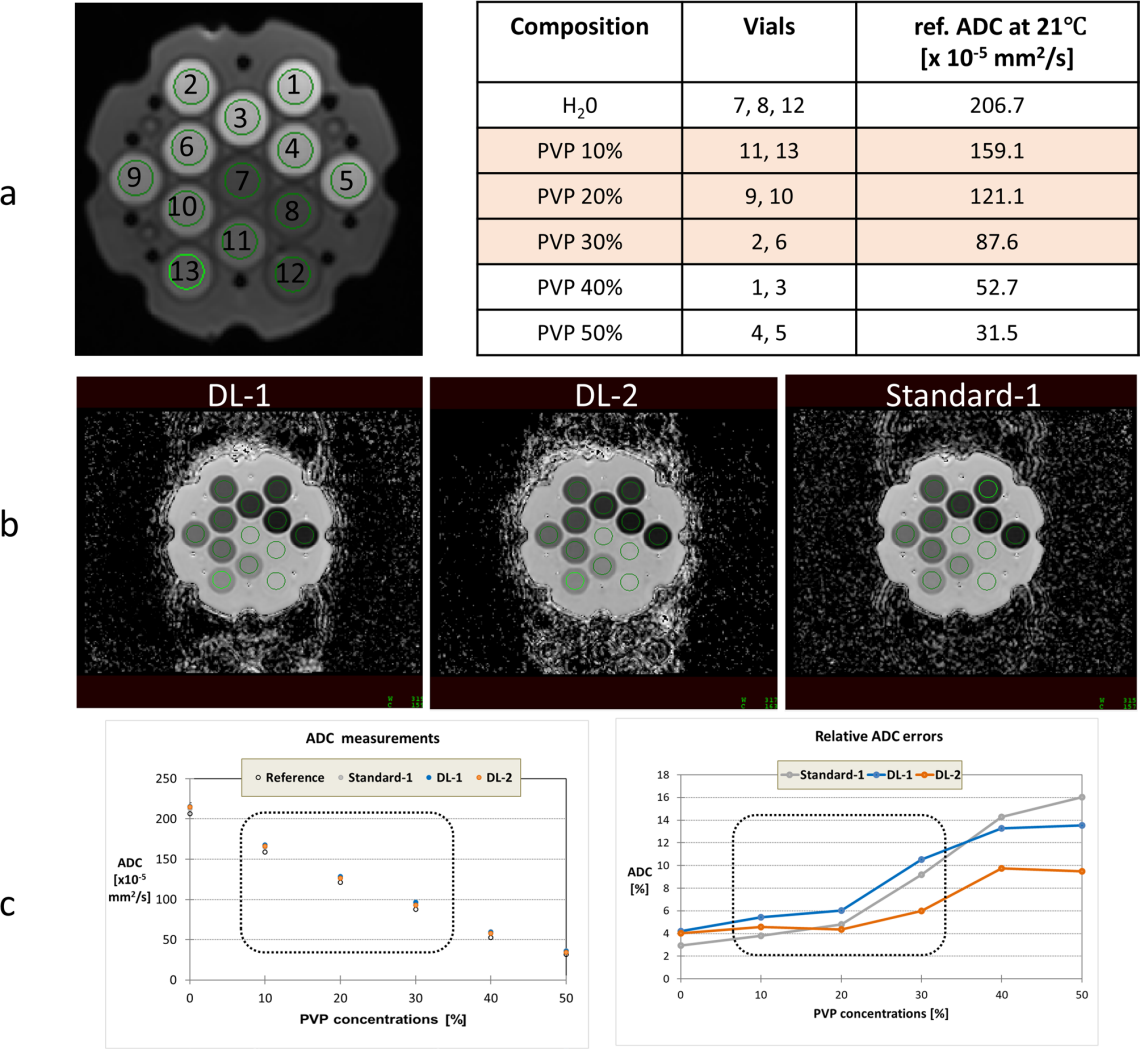
Test-object structure and vials composition (**a**), ADC maps derived from the three DWI series (**b**), and corresponding measured ADC values and their percentual relative error (**c**). Range of ADC values of biological interest is highlighted in cream (panel **a**) and by a rectangle in panel **c.** PVP = polyvinylpyrrolidone; ADC = apparent diffusion coefficient; DL-1 = moderately-accelerated DWI using a deep-learning reconstruction algorithm; DL-2 = highly-accelerated DWI using a deep-learning reconstruction algorithm; Standard-1 = moderately-accelerated DWI using a standard reconstruction algorithm

Two DL-DWI research sequences (Table 2), generating three DWI series, were run on a commercially available test object (NIST/QIBA Diffusion Phantom Model 128, serial number 128–0179, CaliberMRI, Boulder, Colorado) covering a broad range of ADC values (31.5 to 206.7 × 10^− 5^ mm^2^/s at 21 °C). The 13-vial test object (Fig. 2a) is a 19.4-cm spherical phantom containing three vials of pure water and 10 vials of various polyvinylpyrrolidone (PVP) concentrations (two vials per each concentration). The ADC range of clinical interest within this study is mimicked by the 10–30% PVP range. Reference values for all 13 vials were provided by the manufacturer.

**Table 2:** MR parameters for the three compared DWIs, resulted from two acquisitions (DL-1 and DL-2). Parameters that were different between sequences (mostly acquisition acceleration) are shown in bold. Standard-1 data were reconstructed from the DL-1 acquisition using a standard, conventional GRAPPA reconstruction. The DL-1 and DL-2 sequences used the same DL-based reconstruction

DWI Parameters (2D single shot echo planar imaging)	Free-breathing Liver DWI		
	DL-1	DL-2	Standard-1
Acquisition plane	Axial	Axial	Data acquired with the DL-1 method were also reconstructed using conventional GRAPPA reconstruction.
Acquisition time [min: s]	**02:59**	**01:43**	
Voxel size [mm^3^]	1.4 × 1.4 × 5.0	1.4 × 1.4 × 5.0	
TR [ms]	**9500**	**7900**	
TE [ms]	**67**	**63**	
3 b-values [s/mm^2^]	0, 150, 750	0, 150, 750	
Number of averages per b-value	**1, 1, 4**	**1, 1, 2**	
Slices per slab	40	40	
Slice gap [mm]	0	0	
Phase partial Fourier	off	off	
Phase oversampling [%]	30	30	
Matrix (FExPE)	134 × 134	134 × 134	
FOV [mm^2^]	380 × 306	380 × 306	
Interpolation	on	on	
rBW [Hz/Px]	2332	2332	
Parallel-imaging acceleration: PE acc. factor / ref. lines	**2/30**	**3/30**	
Diffusion acquisition	3 scan trace	3 scan trace	
Diffusion encoding scheme	bipolar	bipolar	
Fat suppression	SPAIR	SPAIR	
Reconstruction technique	**DL**	DL	**GRAPPA**

TR = repetition time; TE = echo time; FE = frequency encoding; PE = phase encoding; FOV = field of view; rBW = receiver bandwidth; GRAPPA = generalized autocalibrating partially parallel acquisition; SPAIR = spectral attenuation inversion recovery; DL = deep learning

### MR protocol

This prospective study was performed on a 1.5T MRI scanner (MAGNETOM Sola, Siemens Healthineers, Forchheim, Germany) using a DL-DWI research application package. The liver DWI protocol (Table 2) was a free-breathing axial echoplanar imaging sequence using a 3-direction trace-weighted diffusion encoding, bipolar encoding scheme, 3 b-values (0, 150, 750 s/mm^2^), 40 slices and reconstructed voxel size 1.4 × 1.4 × 5 mm^3^. Based on preliminary healthy volunteer data (see Supplementary Fig. 1), two accelerated sequences (DL-1 and DL-2) were selected to be acquired in patients. The DL-1 sequence used parallel-imaging acceleration factor 2, TE/TR = 67/9500 ms, and 1/1/4 signal averages per b value, acquired in 2:59 min. The DL-2 was a faster sequence, acquired in 1:43 min using an acceleration factor 3, a reduced TE/TR = 63/7900 ms, and 1/1/2 averages per b value.

The same type of DL reconstruction [14] was used for both DL-1 and DL-2 series. The DL reconstruction was based on a variational network, applying data consistency steps and regularization steps in an alternating fashion [14]. In addition to the acquired k-space data, the model also takes precomputed coil sensitivity maps as input. The network architecture comprises six unrolled pre-iterations, each consisting of a data-consistency step using gradient descent and a Nesterov extrapolation step, both with learnable step sizes. These are followed by 11 further unrolled iterations that include the same components, along with an added convolutional neural network (CNN)-based regularization module with hierarchical down-up structure.

Using a complex-valued L1 loss function, the network was trained on about 500,000 individual single-shot DW images which were acquired in volunteers using different clinical 1.5T and 3T scanners (MAGNETOM systems, Siemens Healthineers, Forchheim, Germany). While training was performed offline in PyTorch on an NVIDIA Tesla V100-SXM2 GPU cluster, image reconstruction was executed directly on the scanner via an integrated C++ based inference framework. After reconstruction, standard post-processing steps, including averaging, trace-weighting, and ADC calculation, were applied identical to conventional DWI processing.

Three DWI series were reconstructed out of the two DWI acquisitions. Only the moderately-accelerated acquisition (DL-1) was also reconstructed with conventional GRAPPA (Standard-1) for direct comparison. Preliminary tests of standard reconstruction of the highly-accelerated acquisition (DL-2) demonstrated poor quality images; therefore, standard reconstruction for the DL-2 sequence was not performed for the cohort study.

### Image analysis

#### Qualitative analysis

**Fig. 3:**
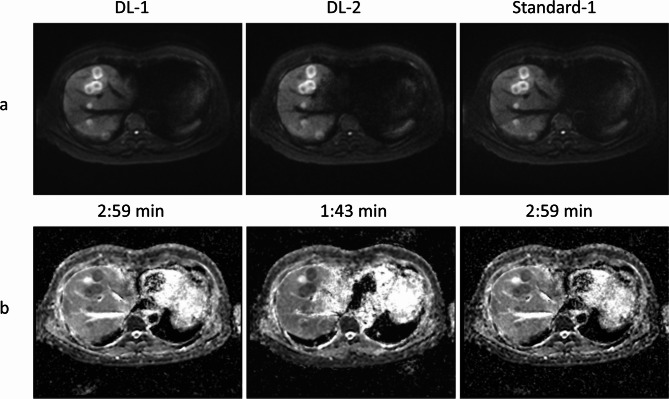
Example of b = 750 s/mm^2^ images (**a)** and ADC maps (**b**) from a 78-year-old male participant demonstrating multiple liver metastases. Contrast display per all b750 images and per ADC maps was kept identical. ADC = apparent diffusion coefficient; DL-1 = moderately-accelerated DWI using a deep-learning reconstruction algorithm; DL-2 = highly-accelerated DWI using a deep-learning reconstruction algorithm; Standard-1 = moderately-accelerated DWI using a standard reconstruction algorithm

Image quality of all three series was assessed independently by two blinded radiologists [JS and FC with 10 and 6 years of experience]. Four features were scored on b750 image (perceived signal-to-noise ratio, sharpness, lesion conspicuity and overall quality) and one on ADC map (overall quality) using a four-point Likert scale (1 = unacceptable/non-diagnostic, 2 = adequate, 3 = good, 4 = excellent); see images/maps example in Fig. 3. All readings were performed on a PACS workstation (Sectra, IDS7, version 20, Sweden) and images were presented in a random order to the radiologists. Cohort median scores for each feature were calculated per series and per reader. No inter-reader agreement assessment was performed.

#### Quantitative analysis

**Fig. 4:**
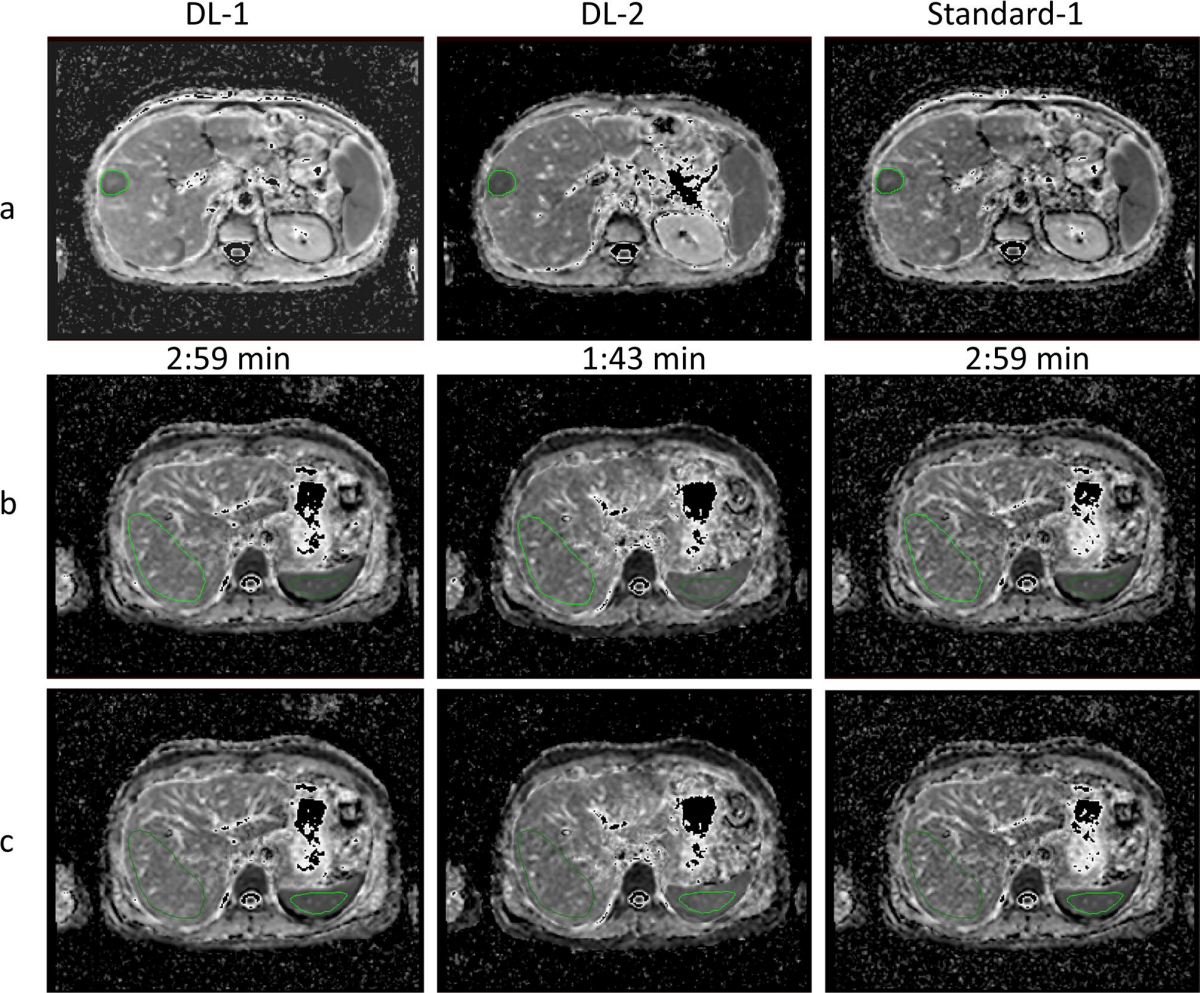
ROI delineation of the three anatomical locations from two different participants: a 40-year-old male participant (**a**) and a 47-year-old female participant (**b**,** c**) Liver metastasis (**a**), normal liver parenchyma (**b**), and spleen (**c**). For each patient, ROIs were matched across the three ADC maps derived from the two DWI acquisitions. Contrast display of all ADC maps was kept identical; ROIs for panel b and c are drawn on the same slice. ADC = apparent diffusion coefficient; DL-1 = moderately-accelerated DWI using a deep-learning reconstruction algorithm; DL-2 = highly-accelerated DWI using a deep-learning reconstruction algorithm; Standard-1 = moderately-accelerated DWI using a standard reconstruction algorithm

Region of interest (ROI) based ADC for all three series were measured (where present) at three locations: liver metastasis, liver and spleen. Volumetric tumour ADC (averaged over multiple slices; slice range 1–9) was measured per participant. Metastases were included if they had a diameter ≥ 2 cm and were located in an area minimally affected by motion (i.e. avoiding left lobe); a maximum of one metastasis was included per participant (Fig. 4a). ROIs were drawn on the highest b-value image and translated to the ADC map. ADC measurements in liver and spleen were derived from a single slice that allowed ROI delineation for both organs within the same slice, in an area that was least impacted by motion, large blood vessels or tumoral tissue (Fig. 4b, c). Participant-specific ROIs were matched across the three ADC maps for each organ location and median ADC were reported. ROI size was kept constant across the three ADC maps for each participant.

**Fig. 5:**
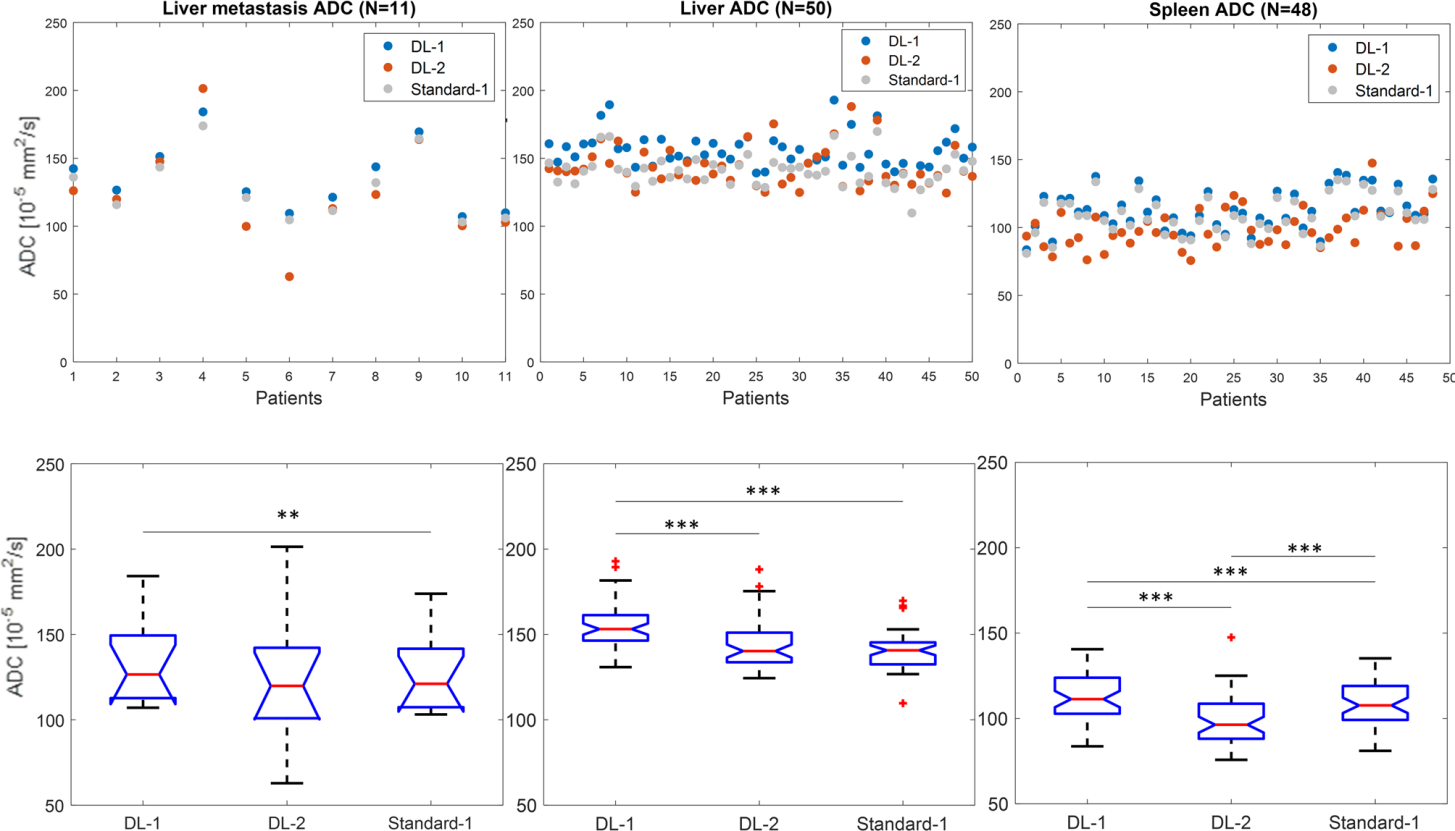
Plots and boxplots of the median ADC for each method measured across liver metastasis, liver and spleen. ** = p-value < 0.01 and *** = p-value < 0.001. Boxplot legend: red central line = median; blue line limits = 25% and 75% percentiles; black line limits = most extreme datapoints that are not outliers; red cross = outlier. Notches are introduced to ease visual comparison between groups: non-overlapping notches suggest statistically significant different medians between groups. Inverted notches visible for metastasis cohort suggest a larger confidence interval for the median value than the quartile. The same vertical scale is displayed for all graphs. ADC = apparent diffusion coefficient. DL-1 = moderately-accelerated DWI using a deep-learning reconstruction algorithm; DL-2 = highly-accelerated DWI using a deep-learning reconstruction algorithm; Standard-1 = moderately-accelerated DWI using a standard reconstruction algorithm

#### Test-object analysis

Circular 146-pixel ROIs were delineated within each of the 13 vials (Fig. 2b) and median ROI-based ADC were reported per vial for each method. ADC values across vials of same composition were averaged and compared against tabulated references; percentual relative measurement errors were reported.

### Statistics

Cohort median results for both qualitative and quantitative analysis (images scores per feature, and ADC per anatomical location) were compared across the three DWI series using the Friedman test (Matlab, R2019a, MathWorks, Natick, MA), and post-hoc analysis (pairwise Wilcoxon tests with Bonferroni correction). A p-value < 0.05 was considered statistically significant.

## Results

### Participants

The study cohort included 50 consecutive participants (mean age 62 years, 26 M) previously diagnosed with colorectal cancer, independent of their past or current treatment status. Note that only some participants had liver metastases, as this criterion was not a prerequisite for enrolment (Fig. 1**)**. No participants were excluded from the final analysis.

All 50 participants were successfully imaged, and 11/50 participants had hepatic metastases corresponding to the criteria defined within the previous section; two patients did not have a spleen because of prior splenectomy.

Qualitatively, the cohort median scores were either good (3) or excellent (4) for all methods (Table 3). The assessment of four image features of the b750 image found that the DL-1 method received an excellent score (4); this was found for 5 out of the total 8 features scored across both readers. Both Standard-1 and DL-2 had a median score of 3, but the interquartile range was lower for the DL-2 method (range 2–3 for 6/8 features) than the Standard-1 (same 2–3 range was found only for 2/8 features). On ADC maps, all three methods scored similarly for each reader; however, a slight discrepancy between the two readers was observed (median scores of 3 from reader 1, and median scores of 4 from reader 2). Overall, DL-1 method was better than the DL-2 method for 7/8 features assessing the b750 image (*p* < 0.001). Similarly, DL-1 was better than the Standard-1 method for 5/8 features. The faster sequence (DL-2) reduced the acquisition time from 2:59 to 1:43 min but exhibited the lowest image quality.

Quantitative results of the cohort ADCs across the three anatomical locations and three series are presented in Table 4, together with the volume of the ROIs and statistics results of the post-hoc analysis. Figure 5 presents plots and boxplots of cohort ADCs, showing higher ADC derived from DL-1 method (blue marker) than that derived from the Standard-1 method (grey marker) across all three investigated locations (p < 0.01). ADC derived from the faster acquisition (DL-2 method, orange marker) was significantly lower than the ADC from DL-1 method in the spleen and liver (p < < 0.001), but not different in liver metastases (p = 0.07)

**Table 3:** Summary of cohort scoring (2 readers, 5 features, 3 DWI series) and their statistics. The Bonferroni correction (for the post-hoc analysis) was implemented by multiplying the p-values by 3 (as there are 3 methods to be compared); therefore, the reported p-values can be higher than 1. All significant p-values (*p* < 0.05) are shown in bold. The post-hoc analysis was not performed for one feature as the p-value of the corresponding Friedman test did not reach statistical significance

Reader 1							
Image quality scoring 4-point Likert scale: 1 = non-diagnostic; 2 = adequate; 3 = good; 4 = excellent.	**Cohort Score**			***p values***			
	*Median (25%-75% quartiles)*			*Friedman*	*post-hoc analysis: Wilcoxon signed rank + Bonferroni correction*		
b750	*DL-1*	*DL-2*	*Standard − 1*		*DL-1 vs. DL-2*	*DL-1 vs. Standard-1*	*DL-2 vs. Standard-1*
Liver SNR	4 (3–4)	3 (2–3)	3 (3–3)	**< 0.001**	**< 0.001**	**< 0.001**	**< 0.001**
Edge sharpness	3 (3–3)	3 (3–3)	3 (3–3)	0.19	x	x	x
Focal lesion conspicuity	3 (3–3)	3 (2–3)	3 (3–3)	**< 0.001**	**< 0.001**	3.00	**< 0.001**
Overall image quality	3 (3–4)	3 (2–3)	3 (3–3)	**< 0.001**	**< 0.001**	**< 0.001**	**< 0.001**
ADC							
Overall image quality	3 (3–3)	3 (2–3)	3 (3–3)	**0.002**	0.06	3.00	0.06
Reader 2							
Image quality scoring 4-point Likert scale: 1 = non-diagnostic; 2 = adequate; 3 = good; 4 = excellent	Cohort Score			*p values*			
	*Median (25%-75% quartiles)*			*Friedman*	*post-hoc analysis: Wilcoxon signed rank + Bonferroni correction*		
b750	*DL-1*	*DL-2*	*Standard − 1*		*DL-1 vs. DL-2*	*DL-1 vs. Standard-1*	*DL-2 vs. Standard-1*
Liver SNR	4 (4–4)	3 (2–3)	3 (2–3)	**< 0.001**	**< 0.001**	**< 0.001**	1.80
Edge sharpness	4 (4–4)	3 (2–3)	3 (3–3)	**< 0.001**	**< 0.001**	**< 0.001**	0.22
Focal lesion conspicuity	4 (4–4)	4 (3–4)	4 (4–4)	**< 0.001**	**< 0.001**	3.00	**0.002**
Overall image quality	4 (4–4)	3 (2–3)	3 (2–3)	**< 0.001**	**< 0.001**	**< 0.001**	0.12
ADC							
Overall image quality	4 (3–4)	4 (3–4)	4 (4–4)	**0.009**	0.07	3.00	0.046

DL-1 = moderately-accelerated DWI using a deep-learning reconstruction algorithm; DL-2 = highly-accelerated DWI using a deep-learning reconstruction algorithm; Standard-1 = moderately-accelerated DWI using a standard reconstruction algorithm; SNR = signal-to-noise ratio; ADC = apparent diffusion coefficient

**Table 4:** Summary of cohort ADC measurements across three methods and three anatomical locations. The volumes of ROIsused for ADC measurements are also presented. The Fridman test was followed by a post-hoc analysis (Wilcoxon signed rank) with a Bonferroni correction where the p-values were multiplied by 3 (as there are 3 methods to be compared); therefore, the reported p-values can be higher than 1. All significant p-values (*p* < 0.05) are shown in bold. sd = standard deviation; ADC = apparent diffusion coefficient

Organ	Cohort size	Parameter	ADC [ x 10^− 5^ s/mm^2^]			Volume [mL]	p values of post-hoc analysis		
			**DL-1**	**DL-2**	**Standard-1**		DL-1 vs. DL-2	DL-1 vs. Standard-1	DL-2 vs. Standard-1
Liver metastasis	11	**median**	**126.6**	**119.9**	**121.1**	**12.0**	0.07	**0.003**	1.24
		mean	135.5	123.7	128.4	27.4	x	x	x
		sd	25.3	36.9	24.2	41.3	x	x	x
Liver	50	**median**	**153.2**	**140.3**	**140.7**	**20.4**	**< 0.001**	**< 0.001**	0.42
		mean	155.7	143.6	140.8	21.8	x	x	x
		sd	12.7	14.5	11.1	10.0	x	x	x
Spleen	48	**median**	**111.3**	**96.3**	**107.6**	**7.5**	**< 0.001**	**< 0.001**	**< 0.001**
		mean	113.2	99.1	109.2	8.5	x	x	x
		sd	14.6	14.5	14.0	4.7	x	x	x

#### Test object

Quantitative results of the test object demonstrated a decrease in accuracy of ADC measurements with increasing PVP concentrations, see Fig. 2c. Overall, the maximal measurement of relative ADC% error was 16% and was found for vials with a 50% PVP. For a clinical range of ADCs (i.e. 10–30% PVP), the maximal ADC error was 10.5% across all series. Similarly to the clinical cohort, the ADCs measured using the DL-2 series (orange line) were lower than ADC derived from the DL-1 series.

## Discussion

This study demonstrates that DL-DWI in the liver can reduce acquisition time whilst maintaining good image quality. The DL-1 method was acquired in 2:59 min versus DL-2 in 1:43 min. Both DWI sequences were based on a clinical sequence acquired in free breathing using a 3-scan trace with 3 b-values with reduced signal averages at the highest b-value.

On b750 images, the moderately-accelerated acquisition (DL-1 method) performed best in terms of SNR, sharpness, lesion conspicuity and overall image quality, whilst the highly-accelerated acquisition (DL-2 method) scored lowest. Note, however, that although image scores for DL-2 were lowest, they were still considered of good quality. Our results suggest that, although the denoising performed well, a reasonable number of averaged acquisitions are required at b750 (i.e. at least 4) to ensure excellent signal for these images for diagnostic use. The high quality of high-b-value images is important as it supports confident identification and accurate evaluation of disease extent by facilitating lesion detection, which may directly impact on the clinical management of patients. The similar scores of all methods on ADC maps suggest no significant qualitative impact of DL reconstruction on ADC maps. Our findings are in line with previous observations showing the superiority of DL-DWI versus a standard non-DL approach, implemented on MR systems from the same vendor [10, 12] and another vendor [9, 11].

Measured ADC values were significantly different between DL-1 and standard reconstructed series when measured across three areas (liver metastasis, liver and spleen), with DL-1-based ADC being higher. Although statistically significant, the relative differences of DL-1-based ADC compared with Standard-1 were still small: 4.5%, 8.9% and 3.4% for liver metastasis, liver and spleen. Such small difference may not be clinically relevant as they fall within the known range of measurement repeatability of ADC [15]. Recently, the Quantitative Imaging Biomarkers Alliance (QIBA) suggested a threshold of 27% repeatability coefficient of ADC in liver lesions [15], which is much higher than the relative differences in ADC reported in our study. The QIBA guidance (derived from review of recent literature) suggest that any treatment-related ADC changes higher than 27% are true changes with 95% confidence. Typically, a threshold at least equal to the repeatability threshold, as seen in [16], is also used as a cut-off for differentiating between responders and non-responders. The heterogeneity of the normal-appearing liver tissue (assessed over a large volume of interest that included small blood vessels) might further explain the ADC variability found for the liver location relative to the other tissues.

A small, negative relative difference (-1.0%, -0.3%, -10.5% for liver metastasis, liver and spleen) was found for the DL-2 series compared with Standard-1; only the difference in spleen measurements was statistically significant. Note, however, that these two ADC series were derived from two separate acquisitions, and motion may be a confounding factor (in particular for ROIs in the spleen). Similarly, a negative relative difference of ADC was found for DL-2 vs. DL-1 series (-5.3%, -8.4%, -13.5% for liver metastasis, liver and spleen), with the liver and spleen measurements being statistically significant. However, any comparison of ADC measurements from DL-2 versus the other two methods should consider the impact of separate acquisitions and the slight difference in other MR parameters (TE, TR) between the two experiments.

Overall, one of the main observations in our clinical cohort (DL-1-based ADC being higher than those derived using DL-2) was also shown on our test-object experiment. As both DL-1 and DL-2 series used the same DL reconstruction network, our results suggest that the observed variations of ADCs might be rather attributed to differences in acquisition parameters than the use of the DL method itself. In addition, the relative difference of DL-1-based ADC compared with Standard-1 in the metastases (4.5%) is similar in size to the difference observed in the phantom (~ 1.5% for the PVP concentrations covering the clinical range of ADCs, i.e. 10–30% PVP).

To our knowledge, there are only a few studies comparing the quantitative ADC values between non-DL and DL DWI [10, 11, 13]. This study assessed DL-DWI reconstruction in the context of a free-breathing acquisition only, without any direct comparison with other motion compensation strategies. The DL reconstruction presented here did not compensate for motion and further motion compensation methods may be needed. Three recent papers [10, 11, 13] investigated DL in the context of DWI with respiratory control. Of these, two papers investigated DL reconstruction with respiratory-triggered DWI [10, 11], whilst another directly assessed DL-DWI in the context of different motion compensation strategies [13]. Recent reports [10, 11] found that DL-ADC was smaller than standard ADC, in agreement with our results for the highly-accelerated DL-DWI series (DL-2). Both reports used a relatively high acceleration factor of 4 [10] or 2.5 [11], similar to the factor 3 used for the DL-2 method in the present study. Conversely, for a lower acceleration factor (i.e. 2, as the DL-1 method within our study), this behaviour was not observed. At present, no other reports assessing a moderately-accelerated DL-DWI are available to compare with our findings. Nevertheless, such small variation of ADCs from its standard value (with either positive or negative bias) may not be clinically relevant. Moreover, Kim et al. [13] found no difference between ADCs measured with/without DL in free-breathing DWI versus respiratory-triggered DL-DWI.

Our study has several limitations. First, the study results were derived from 50 patients diagnosed with colorectal cancer recruited at a single institution. This may limit the generalizability of our findings, especially with regards to other vendor equipment/software, different DWI protocols or patient demographics. Second, all DWI series were acquired in free breathing, which can impact measurement accuracy, in particular for the left liver lobe (widely affected by cardiac and respiratory motion). Therefore, we avoided drawing ROIs for ADC estimates in the left hepatic lobe. On the other hand, any results comparing DL-1 and Standard-1 methods are very robust as both series derived from the same acquisition, i.e. bearing exactly the same impact of respiratory/cardiac motion. In addition, the comparison for liver metastasis ADCs derives from 11 patients only. In the absence of further specific studies, it is not certain that these findings can be translated to non-metastatic liver pathologies (e.g. primary liver tumours or benign lesions).

At present, DL reconstruction methods are available for DWI sequences from all main MR scanner manufacturers at both 1.5T and 3T. For future directions, DL reconstruction could be also applied to other MR sequences such as T1 or T2-weighted imaging that could potentially decrease the total scanning time for a liver MR protocol even more.

## Conclusions

This study suggest that a DL-based reconstruction using a variational network is a promising technique to enable acceleration of liver DWI considering both qualitative and quantitative results. The DL-based reconstruction approach can be used in two different ways, depending on the clinical application: a moderately-accelerated DL approach (DL-1) could maintain image quality whilst still offering an acceptable acquisition scan time. Otherwise, in cases where very fast scans are required, a highly-accelerated DL approach can provide shorter acquisitions with a good image quality.

Quantitatively, small ADC differences were found between DL and non-DL DWI, but these may not be considered clinically significant.

## Supplementary Information

Below is the link to the electronic supplementary material.

Supplementary Material 1: Fig. 1 Healthy volunteer experiment that tested acceleration factors (2 to 7) and averages per b-value (112 vs 114; i.e. 1 average for b0, 1 average for b150 and 2 or 4 for b750 s/mm2). Both rows display b750 images. Top row: same averaging scheme (112) was kept for all acceleration factor tests; the arrow shows ghosting artefacts appearing at higher acceleration factors.

## Data Availability

The datasets generated and/or analysed during the current study are not publicly available due to reasons of sensitivity but are available from the corresponding author on reasonable request.

## Abbreviations

ADC: Apparent diffusion coefficient
DL: Deep learning
DWI: Diffusion weighted imaging
PVP: Polyvinylpyrrolidone
ROI: Region of interest
SNR: Signal to noise ratio
TE: Echo time
TR: Repetition time
