# Supplementary figures and images for: Qualitative and quantitative assessment of accelerated liver diffusion-weighted imaging using deep-learning reconstruction in oncologic patients
Source: BMC Med Imaging. 2025 Nov 26;25:491. doi: 10.1186/s12880-025-02030-3 (PMC12659593; doi:10.1186/s12880-025-02030-3)

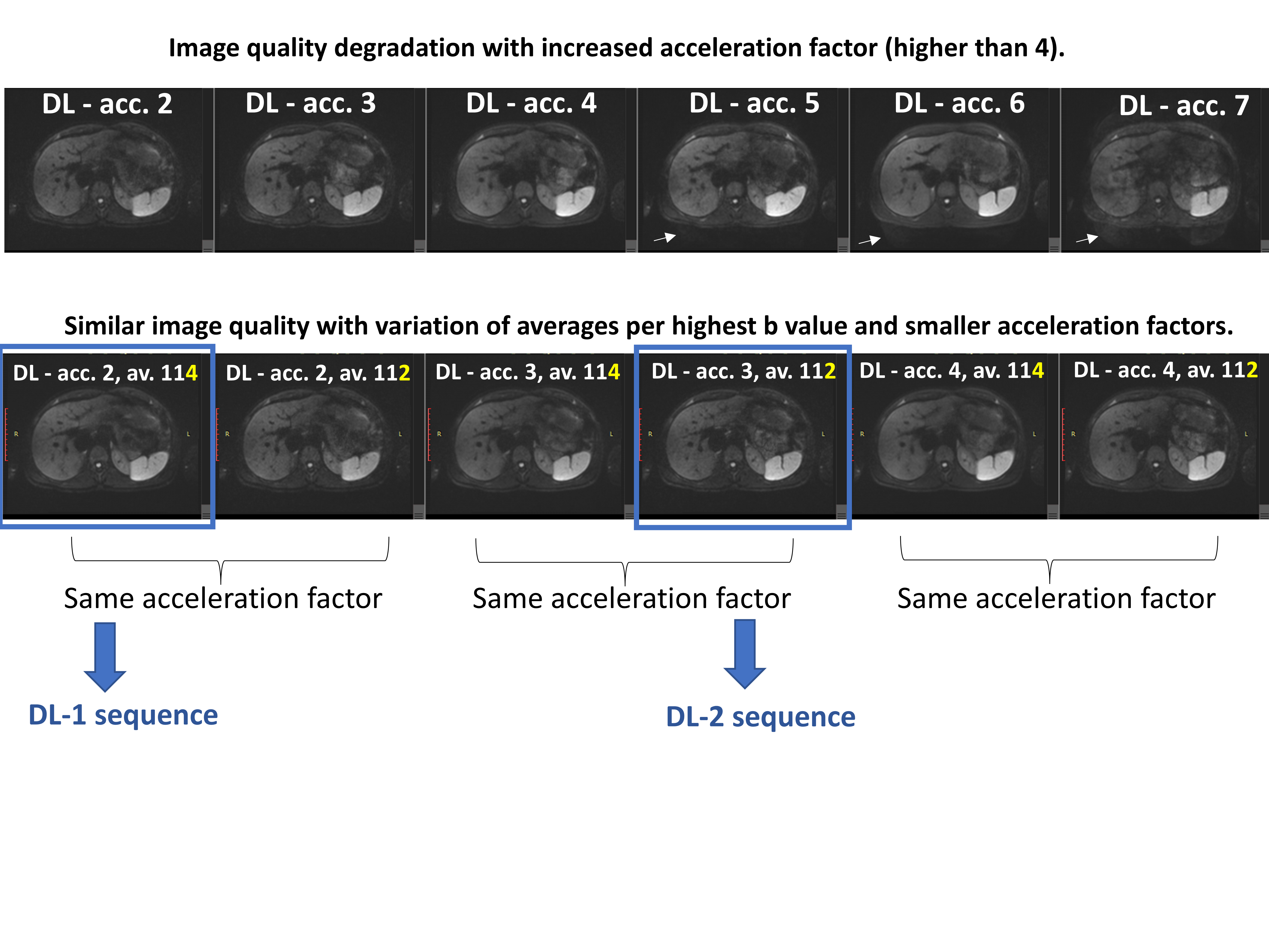

Supplement: Supplementary file 1 — Supplementary Material 1: Fig. 1 Healthy volunteer experiment that tested acceleration factors (2 to 7) and averages per b-value (112 vs 114; i.e. 1 average for b0, 1 average for b150 and 2 or 4 for b750 s/mm2). Both rows display b750 images. Top row: same averaging scheme (112) was kept for all acceleration factor tests; the arrow shows ghosting artefacts appearing at higher acceleration factors. [file 12880_2025_2030_MOESM1_ESM.tif]
